# Spatial transcriptomic profiling reveals body site-specific inflammatory differences in psoriasis lesions

**DOI:** 10.3389/fimmu.2026.1706701

**Published:** 2026-03-12

**Authors:** Thomas Emmanuel, Hakim Ben Abdallah, Morten Muhlig Nielsen, Borislav Ignatov, Elena Baez, Anna Skarnvad Andersen, Line Waaben, Mette Boye, Ida Kaaber, Desirée Sofie Boonen, Søren Riis Petersen, Torben Steiniche, Anne Bregnhøj, Liv Eidsmo, Lars Iversen, Christian Vestergaard, Claus Johansen

**Affiliations:** 1 Department of Dermatology, Aarhus University Hospital, Aarhus, Denmark; 2 Department of Clinical Medicine, Aarhus University Hospital, Aarhus, Denmark; 3 Department of Molecular Medicine, Aarhus University Hospital, Aarhus, Denmark; 4 Department of Medicine, Karolinska Institutet and PO Reuma/Hud/Gastro, Karolinska University Hospital, Stockholm, Sweden; 5 LEO Foundation Skin Immunology Research Center, University of Copenhagen, Copenhagen, Denmark; 6 Department of Pathology, Aarhus University Hospital, Aarhus, Denmark

**Keywords:** immunohistochemistry, psoriasis, RNA sequencing, spatial transcriptomics, treatment-resistance

## Abstract

**Introduction:**

Psoriasis is a common chronic inflammatory skin disease. Treatments lead to Q6 substantial improvement of most psoriasis plaques. However, it can be challenging to reach disease resolution in certain hard to treat areas such as scalp, and lower extremity. Here we map histologic and spatial transcriptomic differences between psoriasis lesions across different anatomical locations, to understand if differences can be linked to plaque-site specific treatment resistance.

**Methods:**

Quantitative immunohistochemical analysis and transcriptomic digital spatial profiling were performed on skin punch biopsies obtained from unaffected areas on the trunk, lesional (LS) areas of the scalp, upper extremity and lower extremity of 12 patients with psoriasis. Histological analysis showed no significant differences in epidermal thickness among LS skin from different body locations.

**Results:**

Immunohistochemical markers (CD3, CD4, CD8, CD103, CD207, IL-12RB1, IL-17A, IL-23R, RORγt, FOXP3, and MPO) did not differ significantly between LS sites. Whole transcriptome spatial RNA profiling identified several differentially expressed genes that revealed site-specific transcriptomic differences. Notably, IL-23 signaling was significantly enriched in the lower extremity epidermis, and IL-17 signaling was more pronounced in the epidermis of LS samples.

**Discussion:**

These findings highlight minimal histological and immunohistochemical variation, yet significant transcriptomic and pathway differences between psoriasis body locations, suggesting potential targets for site-specific therapeutic strategies.

## 1 Introduction

Psoriasis is a common, chronic, inflammatory skin disease that significantly impacts the quality of life and overall health of patients (1). Psoriasis exhibits a wide range of clinical presentations, with plaque psoriasis being the predominant type. Plaque psoriasis is characterized by distinct erythematous, infiltrated patches on the skin, with dry, silver-like scales. These lesions are commonly found on the scalp, torso, and extensor surfaces of the extremities (1–3). The pathogenesis behind psoriasis is multifaceted and is not yet fully elucidated. However, psoriasis is considered a T-cell mediated disease that relies on communication between the body’s innate and adaptive immune responses, in which dendritic cells and keratinocytes are crucial players (4, 5). There are several treatment options for psoriasis ranging from topical therapy, phototherapy, and climatotherapy, to oral systemic therapies, and biologics. The choice of treatment depends on various factors including severity of disease and individual patient preferences (1, 6). Despite the current wealth of treatment options and even after long-term disease-free remission, some patients experience reccurrence of psoriasis at previously affected areas, indicating a form of disease memory (7, 8).

The exact mechanisms underlying this disease memory are not yet fully understood. However, studies have revealed the existence and activation of pathogenic tissue-resident memory T-cells (TRMs) in non-lesional (NL), lesional (LS), and resolved LS psoriasis skin (9–11).

Additionally, variations in treatment response across different anatomical locations, with certain areas being particularly hard to treat, further challenge the management of psoriasis. Notably, the scalp, face, nails, palms, soles, and genitals are frequently reported as hard-to-treat or special areas (12–14).

Dermoscopic features of psoriatic plaques differ based on the anatomical location (15, 16), and location-dependent DNA methylation patterns in psoriasis afflicted skin has been shown (17). Limitations in the understanding of the heterogeneity of psoriasis lesions in specific anatomical locations may result in suboptimal treatment.

In this study, we investigated body-site specific lesions in psoriasis patients by combining quantitative immunohistochemical analysis and transcriptomic digital spatial profiling (DSP). Using this integrative approach, we aimed to uncover the molecular heterogeneity of psoriatic lesions at different anatomical locations and gain further insight into the differences that may be involved in the observed plaque-site specific treatment resistance.

## 2 Materials and methods

### 2.1 Study design

12 patients with psoriasis, three with atopic dermatitis, and three healthy controls were included in this study. All patients provided written informed consent, and the study was approved by the local ethics committee (number: m-20090102). Patient demographics are provided in **Supplementary Table 1** and **Supplementary Table 2**.

### 2.2 Biopsy retrieval

Four-mm punch biopsy specimens from up to four different body locations were obtained from patients with psoriasis. The anatomical locations of the four biopsies included NL skin from the trunk and LS skin from the scalp, UE, and LE. See **Supplementary Table 3** for specific biopsy locations for each patient. Upon biopsy acquisition, the specimens were formalin fixed and paraffin-embedded. NS and AD samples were acquired from pseudorandomized biobank material where only disease category, gender and age was known from patients. AD samples were collected from clinically active inflammatory lesions.

### 2.3 Histology and quantitative immunohistochemistry

Four-µm thick skin sections from the paraffin-embedded specimens were used. Hematoxylin and Eosin (HE) staining was performed as previously described (18). Prior to immunohistochemical staining, sections were deparaffinized and then rehydrated in a series of graded ethanols. Subsequently, antigen unmasking was accomplished using heated Tris-ethylene glycol tetraacetic acid buffer (pH 9) at pre-boiling temperature. The antibodies used were CD3, CD4, CD8, CD103, CD207, RORγt, FOXP3, Ki67, and MPO. See **Supplementary Table 4** for information regarding dilution, clone, isotype, vendor, and incubation length. For antibody detection, UltraVision Quanto Detection System HRP DAB (cat. TL-060-QHD, Thermo Fisher Scientific Waltham, MA, USA) and MultiVision anti-mouse/HRP + anti-rabbit/AP (cat. TL-012-MHRA, Epredia, Kalamazoo, MI, USA) were used for single and double staining, respectively.

For image analysis, slides were digitalized using the NanoZoomer 2.0-HT slide scanner (Hamamatsu Photonics K.K., Hamamatsu, Japan) with a 20x objective. For quantification of immunohistochemically stained cells, the open-source software QuPath (v.0.4.3) (19) was used as previously described using a combination of pixel classification and counts with an area expanding 500 µm below the ventral part of the epidermis defined as the dermal region of interest (20).

### 2.4 Spatial transcriptomics

To conduct spatial transcriptomics, the sections were mounted on positively charged Superfrost Plus histology microscope slides (cat. 12-550-15, Fisher Scientific, Thermo Fisher Scientific Waltham, MA, USA). The analyses were then performed using the GeoMx DSP technology (NanoString Technologies Inc., Seattle, USA). Slides were blocked for 30 minutes and then incubated for one hour with a cocktail of DSP Barcoded RNA (indexing RNA oligos) and fluorescently conjugated antibodies to PanCK, Vimentin, and DNA (**Supplementary Table 4**). Next, the slides were washed in 2x saline-sodium citrate. Subsequently, the slides were immersed in PBS with 0.1% Tween 20 and placed in the DSP instrument according to the instructions outlined in the NanoString Protocol.

### 2.5 Areas of illumination selection

Fluorescence scans (x20) were first conducted to capture precise images of the tissue prior to AOI selection. A maximum of five AOIs were chosen per tissue, which included the epidermis (300 nuclei), the basal layer of the epidermis (200 nuclei), dermal papillae (200 nuclei), an area with low vimentin concentration (200 nuclei), and an area with high vimentin concentration (200 nuclei). Nuclei identification was conducted using the DNA marker in the GeoMx software and based on recommendations by NanoString.

### 2.6 Library preparation and sequencing

The RNA oligos (probes) that were indexed with a DSP barcode were released from each AOI using ultraviolet light exposure. Subsequently, probes were collected using a microcapillary tip and placed into a 96-well plate. The probes were quantified using Next Generation Sequencing. The resulting read number of each probe was then correlated to the expression level of its gene target. Indexed RNA oligos from each AOI were polymerase chain reaction amplified using primers that (i) hybridized to constant regions and (ii) contained unique dual-indexed barcoding sequences to preserve AOI identity. PCR products were pooled and purified twice with Ampure XP beads. Library concentration and purity were measured using high-sensitivity DNA Bioanalyzer chip. Paired-end (2x75 bp reads) sequencing was performed on an Illumina MiSeq instrument.

### 2.7 Data processing

The GeoMx HGS Pipeline software from NanoString was utilized to generate count data files (dcc files) from NGS fastq files. The R statistical programming language was utilized for subsequent data analysis, along with the following packages: NanoStringNCTools, GeomxTools, and GeomxWorkflows. Fraction of trimmed, stitched and aligned reads were above 80% for all segments. Sequencing saturation was above 90% for all segments.

### 2.8 Normalization

Third quartile (Q3) normalization was used prior to downstream visualization and differential expression analysis. Data were stratified according to location and five separate linear mixed models for the basal layer, vimentin high, vimentin low, epidermis, and dermal papillae were applied for all 4 locations with scan id variable as a random factor. In addition, two models were made to combine evidence across different body locations. GSVA was performed with R software (version 4.2.0) and the GSVA package (version 1.46.0) using Reactome genesets (see **Supplementary Table 5** for gene sets) imported from the Molecular Signature Database through MSigDB (version 7.5.1) (21).

### 2.9 Statistical analysis

Data normality was assumed for immunohistochemistry cell counts. We applied a mixed model analysis with *post hoc* Tukey to compare the different body locations. The statistical analysis excluded missing data. A P-value < 0.05 was considered statistically significant. Volcano plots were used for interpreting differential gene expression results.

## 3 Results

### 3.1 No histological differences were observed between psoriasis skin locations

**Figure 1:**
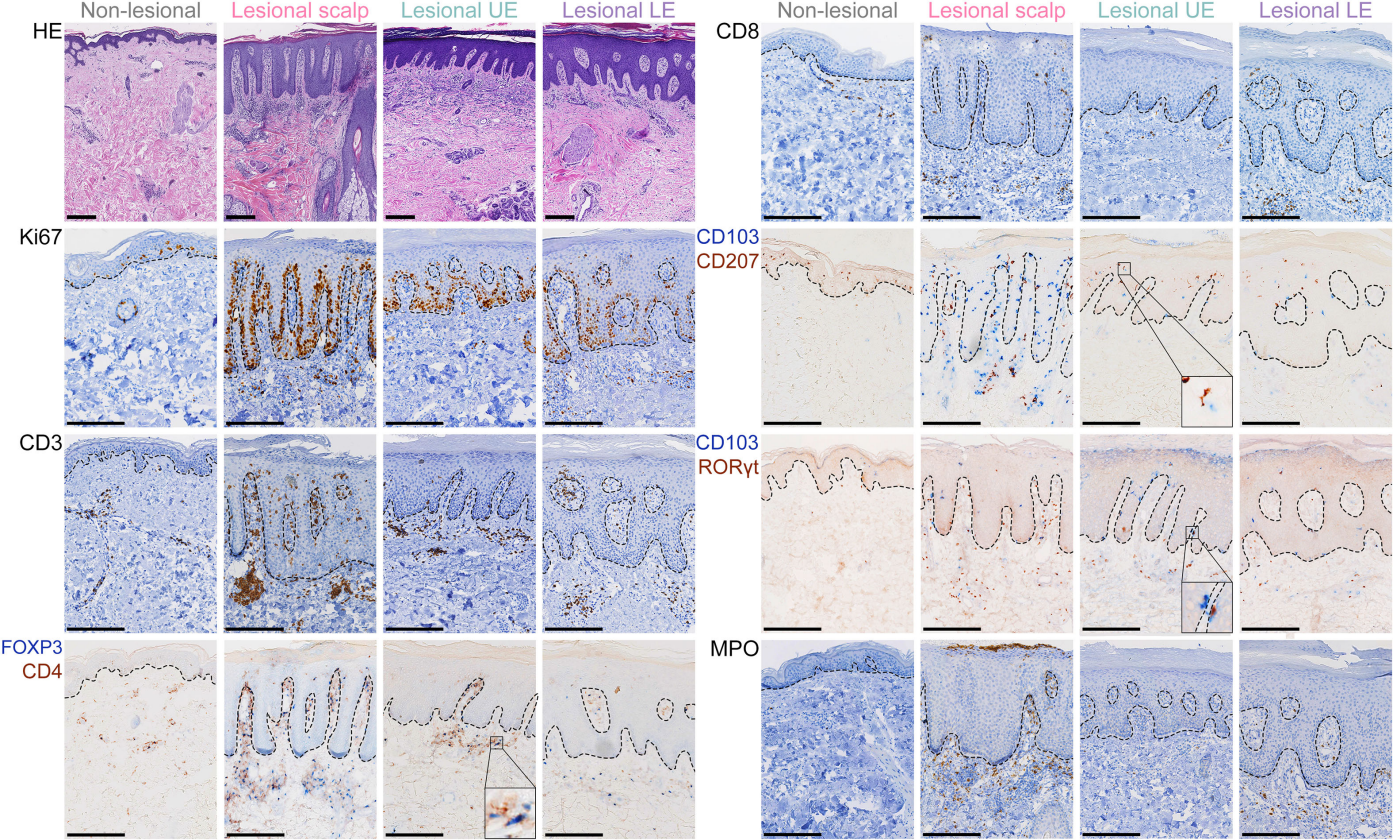
Immunohistochemical staining from four body locations. Examples of hematoxylin and eosin (HE) staining and immunohistochemistry of Ki67^+^, CD3^+^, CD4^+^, FOXP3^+^, CD8^+^, CD103^+^, CD207^+^, RORγt^+^, and MPO^+^ cells from non-lesional skin, lesional psoriasis skin from the scalp, lesional skin from the upper extremity (UE), and lesional skin from the lower extremity (LE). Close-up of the CD4^+^/FOXP3^+^ and CD103^+^/RORγt^+^ panels illustrate examples of single and double positive cells. Closeup of the CD103^+^/CD207^+^ panel illustrates an example of close interaction (touching) of a CD103^+^ and CD207^+^ cell. Size bars = 250 μm.

**Figure 2:**
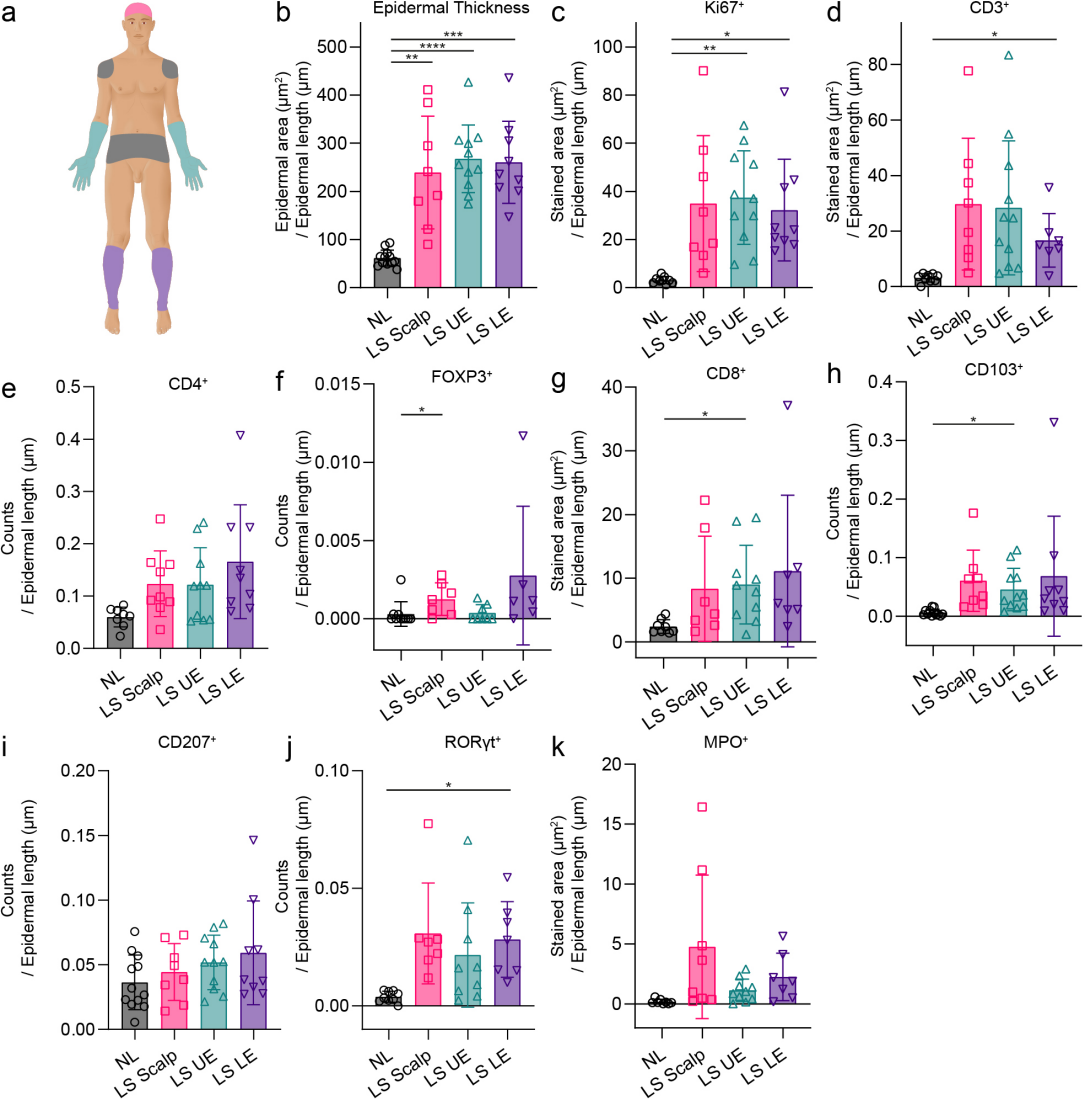
Immunohistochemical results from the different body locations. **(a)** Illustration of anatomical locations where biopsies were obtained: Non-lesional (NL) skin from the trunk (grey), lesional skin from the scalp (LS Scalp) (ruby), upper extremity (LS UE) (mint), and lower extremity (LS LE) (purple). **(b)** Results from hematoxylin and eosin (HE) staining used to quantify the epidermal thickness. **(c-k)** Results from quantitative immunohistochemistry of Ki67^+^, CD3^+^, CD4^+^, FOXP3^+^, CD8^+^, CD103^+^, CD207^+^, RORγt^+^, and MPO^+^ cells from the four body locations. Mean ± SD depicted. *P < 0.05, **P < 0.01, ***P < 0.001, ****P < 0.0001.

First, we evaluated histological variations across different psoriatic anatomical sites, specifically the scalp, upper extremity (UE), and lower extremity (LE) (**Figures 1**, **2**). Epidermal thickness was quantified using HE staining as shown in **Figure 1**.

As expected, the epidermal thickness in LS psoriatic skin was significantly increased compared with NL skin. However, no significant differences in epidermal thickness were observed between LS skin from the different body locations (**Figure 2B**).

Next, we investigated the expression of the psoriasis-related markers CD3, CD4, CD8, CD103, CD207, RORγt, FOXP3, and MPO (**Figure 2** and **Supplementary Figure 1**). Higher numbers of cells were observed in LS skin compared with NL skin, however, none of the investigated psoriasis-related markers revealed significant differences among LS skin from different body locations. An analysis was performed to investigate the co-location and co-interaction of CD4^+^, CD103^+^, CD207^+^, RORγt^+^, and FOXP3^+^ cells in LS skin from different locations. However, no significant differences were observed between any of the psoriasis locations (**Supplementary Figure 1**).

Interestingly, the quantity of CD207^+^ cells did not increase (**Figure 2**) in LS skin, however we observed that a higher percentage of these cells interacted with CD103^+^ cells in LS skin (**Supplementary Figure 1C**). No difference in the interaction between CD207^+^ cells and CD103^+^ cells was observed between LS sites (**Supplementary Figure 1C**).

### 3.2 Whole transcriptome spatial RNA profiling recapitulated known differences between normal skin, atopic dermatitis and psoriasis

**Figure 3:**
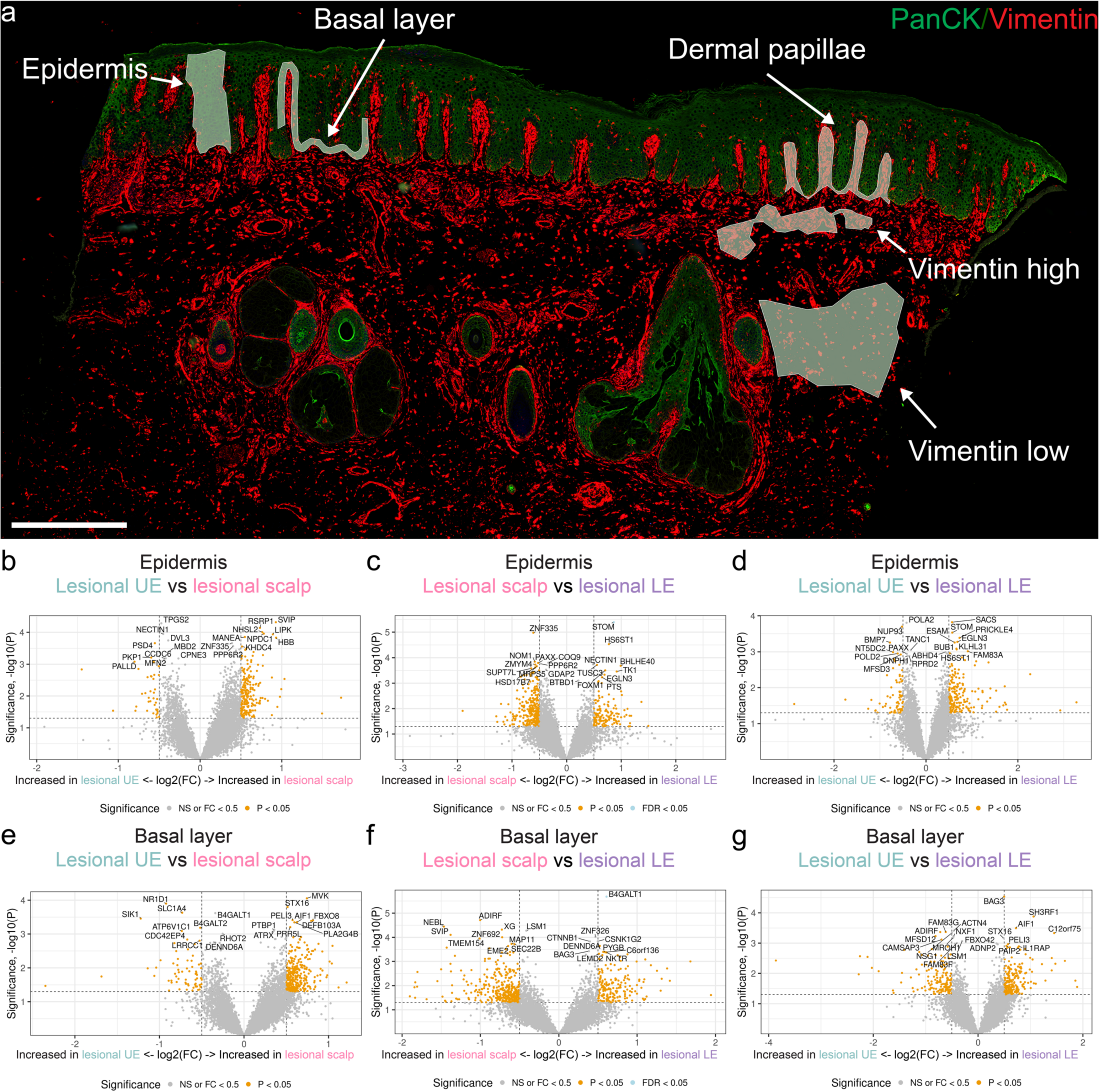
Areas of interest and volcano plot analysis from the different body locations and different areas of interest. **(a)** Examples of areas of illumination selected using GeoMx digital spatial profiling software. Pan cytokeratin (PANCK) is depicted in green and vimentin in red. **(b)** Differentially expressed genes (DEGs) in the epidermis between lesional (LS) scalp and LS upper extremity (UE). **(c)** DEGs in the epidermis between LS scalp and LS lower extremity (LE). **(d)** DEGs in the epidermis between LS UE and LS LE. **(e)** DEGs in the basal layer between LS scalp and LS UE. **(f)** DEGs in the basal layer between LS scalp and LS LE. **(g)** DEGs in the basal layer between LS UE and LS LE. Size bar = 500 μm.

Following the confirmation of immunohistochemical homogeneity across psoriatic lesions, we utilized the GeoMx platform to profile the whole transcriptome of specific areas of interest (AOIs). To validate the platform’s capacity to differentiate distinct dermatological conditions, we initially performed a comparative analysis across healthy control skin (NS), atopic dermatitis (AD), and all psoriasis samples across the various AOIs. To simulate a standard full-thickness skin biopsy, we computationally aggregated transcriptomic data from all distinct AOIs within each biopsy type to generate ‘pseudo-bulk’ samples. This approach allowed for a representative global comparison across the different skin conditions (see **Supplementary Figure 2** for HE staining of NS and AD samples, and **Figure 3A** for a visualization of the selected AOIs).The t-distributed stochastic neighbor (tSNE) plot showed a clear difference between the various distinct AOIs, while differences between diseases and within AOIs were less clear (**Supplementary Figure 3A**). Interestingly, the method recapitulated known differences between the various skin samples (up in AD vs NS e.g. *FAM102A* and *S100* genes), (up in psoriasis vs NS e.g. *FABP5* and *S100* genes), (up in AD vs psoriasis e.g. *CCL27* and down e.g. *IL-36G*) (**Supplementary Figures 3B–D**) (22–24).

### 3.3 Whole transcriptome digital spatial profiling analysis delineated differences between psoriasis body locations

**Figure 4:**
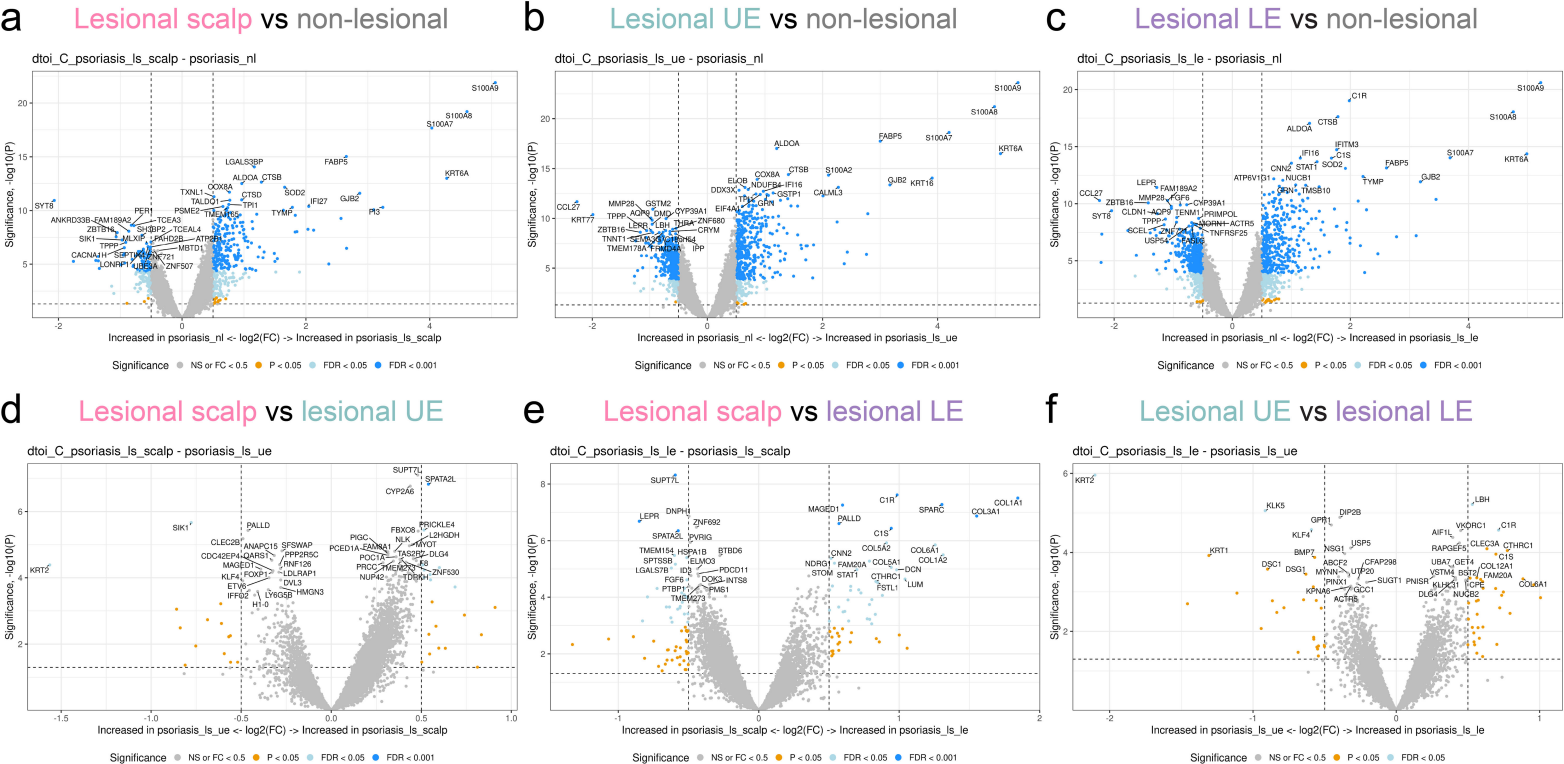
Digital spatial profiling of all areas of interest and psoriasis skin locations. **(a)** Volcano plot depicting the differentially expressed genes (DEGs) between lesional scalp and non-lesional skin of all areas of interest (AOIs) in grouped into one. **(b)** Volcano plot depicting the DEGs between non-lesional skin and lesional upper extremity (UE) and of all AOIs grouped into one. **(c)** Volcano plot depicting the DEGs between non-lesional skin and lesional lower extremity (LE) of all AOIs grouped into one. **(d)** Volcano plot depicting the DEGs between lesional scalp and lesional upper extremity (UE) of all areas of interest AOIs grouped into one. **(e)** Volcano plot depicting the DEGs between lesional scalp and lesional LE of all AOIs in grouped into one. **(f)** Volcano plot depicting the DEGs between lesional UE and lesional LE of all AOIs grouped into one.

Having recapitulated known differences in different skin conditions, we next wanted to compare different psoriasis locations. The differentially expressed genes (DEGs) (eg. *S100A7*, *S100A8* and *S100A9*) clearly separated the three LS biopsies from NL skin thus confirming the presence of a psoriatic phenotype in the biopsies (**Figures 4A, B**). Subsequently, we compared each lesional skin location (**Figures 4D–F**). Several genes were differentially expressed in the scalp compared with UE. *SPATA2L* and *PRICKLE4* were the most upregulated genes in scalp compared with UE while *SIK1* and *KRT2* were found to be the most downregulated (**Figure 4D**). In the comparison between the scalp and the LE, the genes *SUPT7L, LEPR*, and *SPATA2L* showed increased expression in the scalp, whereas *COL1A1, COL3A1, SPARC, C1R, PALLD*, and *MAGED1* displayed decreased expression in the scalp (**Figure 4E**).

We identified significant gene expression differences between the UE and LE; specifically, *KRT2*, *KLK5*, and *KLF4* expression increased in the UE, while *C1R* and *LBH* decreased (**Figure 4F**). This suggests that the psoriatic transcriptome is influenced by anatomical location.

### 3.4 Whole transcriptome spatial RNA profiling from the different body locations and different areas of interest revealed novel differences in psoriasis locations

Having observed that differences exist between the NL and LS skin locations using pseudo-bulking, we analyzed the segments further by comparing the individual AOIs. The chosen AOIs represented areas with functional differences in psoriasis (**Figure 3A**).

**Figure 5:**
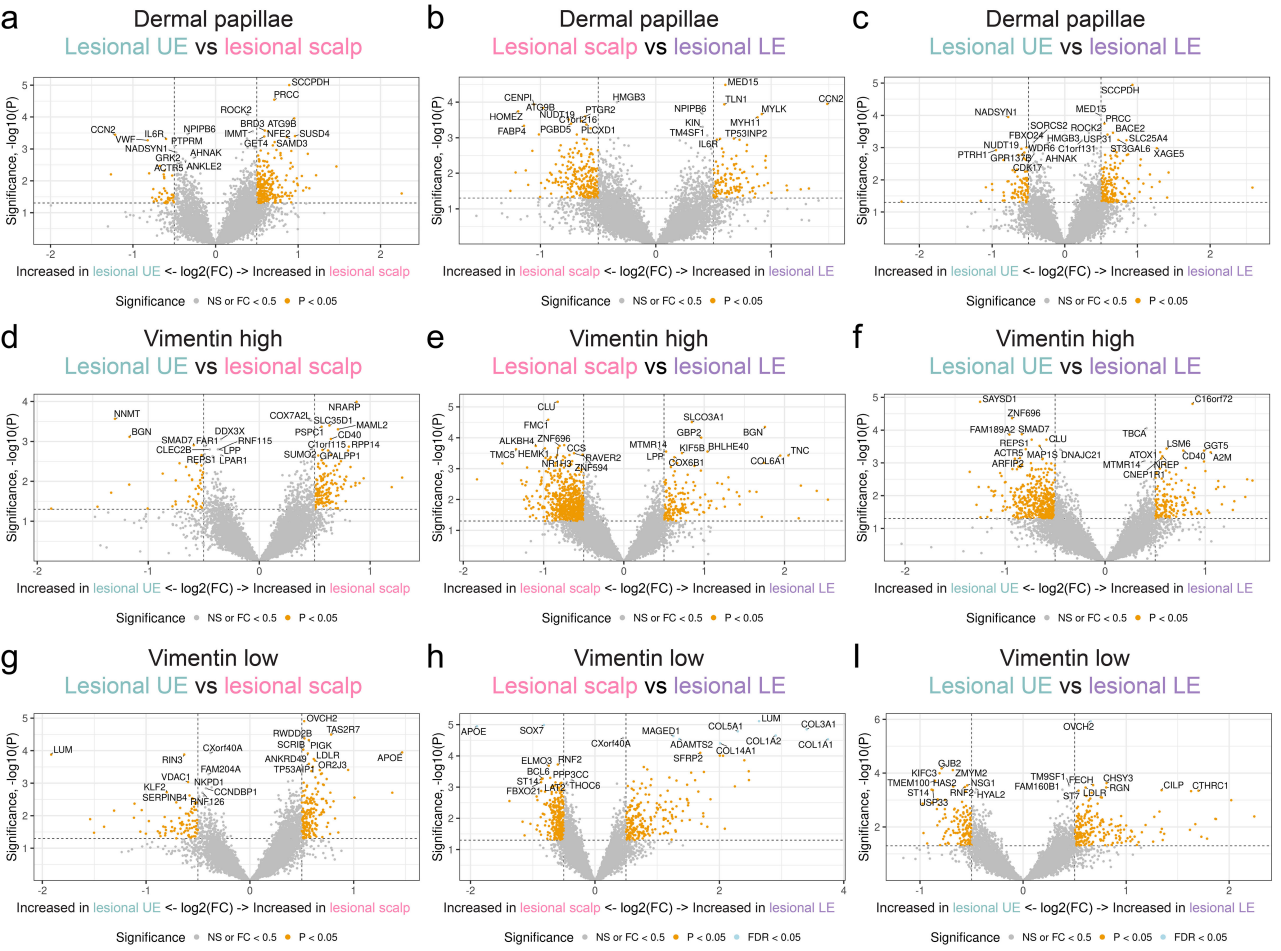
Differential gene expression analysis from the dermal papillae, vimentin high, and vimentin low area from different body locations. **(a)** Differentially expressed genes (DEGs) in the dermal papillae between lesional (LS) scalp and LS upper extremity (UE). **(b)** DEGs in the dermal papillae between LS scalp and LS lower extremity (LE). **(c)** DEGs in the dermal papillae between LS UE and LS LE. **(d)** DEGs in the vimentin high area between LS scalp and LS UE. **(e)** DEGs in the vimentin high area between LS scalp and LS LE. **(f)** DEGs in the vimentin high area between LS UE and LS LE. **(g)** DEGs in the vimentin low area between LS scalp and LS UE. **(h)** DEGs in the vimentin low area between LS scalp and LS LE. **(i)** DEGs in the vimentin high low between LS UE and LS LE.

By focusing only on genes achieving false discovery rate < 0.05, several DEGs were discovered. *STOM* was significantly upregulated in the epidermis in the LE compared with the scalp (**Figures 3B–D**). Furthermore, *B4GALT1* exhibited a significant upregulation in the basal layer of the LE compared with the scalp (**Figures 3E–G**). In addition, we compared the dermal papillae, areas with high vimentin expression, and areas with low vimentin expression between the various biopsy sites (**Figure 5**).

The results indicated that *LUM*, *COL1A1*, *COL1A2*, *COL3A1*, *COL5A1*, *COL14A1*, and *MAGED1* displayed a significant increase in expression in the vimentin low areas of the LE when compared to the scalp, while *APOE* and *SOX7* demonstrated a significant decrease in expression (**Figure 5H**).

### 3.5 Pathway analysis identified differences in IL-17 and IL-23 signaling pathways between different psoriasis locations

**Figure 6:**
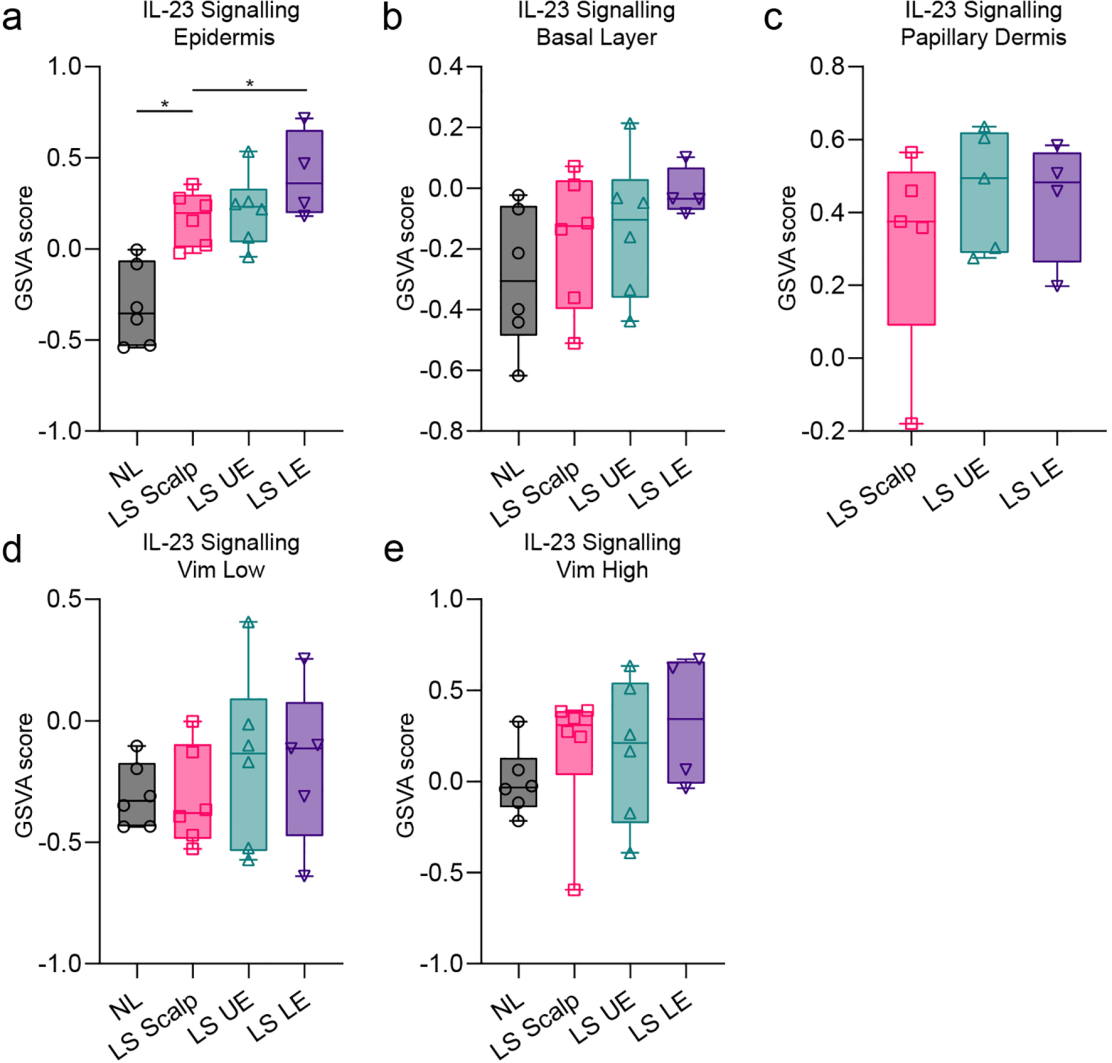
Gene set variation analysis of the IL-23 signaling pathway from the different psoriasis body locations. **(a)** IL-23 signaling in the epidermis. **(b)** IL-23 signaling in the basal layer. **(c)** IL-23 signaling in the papillary dermis. **(d)** IL-23 signaling in the vimentin low area (Vim Low). **(e)** IL-23 signaling in the vimentin high area (Vim High).

Because some focal differences existed in the transcriptome between LS psoriasis locations, we next investigated whether specific pathways were enriched in specific psoriasis locations and in specific AOIs using gene set variation analysis (GSVA). We focused on tumor necrosis factor (TNF), IL-17, and IL-23 signaling pathways because they represent effective therapeutic targets in psoriasis treatment (1). Interestingly, IL-23 signaling was significantly enriched the epidermis of the LE (**Figure 6**). Furthermore, IL-17 signaling was significantly more pronounced in the epidermis of LS psoriasis samples, while being less prominent in the vimentin low and high regions of the dermis, however no differences were observed between the LS locations (**Supplementary Figure 4**).

We also examined TNF signaling, however no significant differences were observed between the biopsy locations (**Supplementary Figure 5**).

### 3.6 No histological differences in IL-12RB1, IL-17A and IL-23R were observed between psoriasis skin locations

Due to location-dependent differences in IL-17A and IL-23 signaling, we assessed IL-17A, IL-12RB1, and IL-23R expression by immunohistochemistry (**Supplementary Figure 6**). Lesional skin showed a trend toward increased expression of all three markers compared to non-lesional controls (**Supplementary Figure 7**). Dermal IL-12RB1 was significantly higher in lesional UE and LE (p < 0.05), while IL-17A and IL-23R were predominantly elevated in the dermis of LS UE and LS scalp. These findings support activation of the IL-12/IL-23–Th17 axis in lesional skin. However, no significant differences were found between lesional locations.

## 4 Discussion

Site-specific treatment resistance is a challenge in psoriasis management. In this study, we aimed to investigate whether histologic or spatial transcriptomic differences exist between four different site-specific areas from patients with psoriasis and whether these could contribute to the observed treatment resistance.

Interestingly, we found that LS areas from the scalp, UE, and LE, displayed a different transcriptomic signature. Furthermore, despite histological similarity, distinct AOIs in the skin revealed different gene expressions across the samples.

A previous study conducted on five psoriasis patients with biopsies from abdomen, arm, back, buttock, leg and thigh showed an individualized inflammatory response between patients while a difference between sites was less pronounced (25).

By combining the AOIs for each location into a pseudo-bulk sample, we found that *SPATA2L* was upregulated in the scalp compared with both the UE and the LE. *SPATA2L* may have a possible role in the pathogenesis of vitiligo (26), but its function in psoriasis remains unclear. *SIK1 and KRT2* were upregulated in the UE compared with the scalp. *KRT2* expression has been shown to correlate with Psoriasis Area and Severity Index (PASI) in LS skin and the expression levels of *KRT2* in NL skin may predict treatment response with etanercept (27), thus suggesting the potential of differential effects of etanercept on different psoriasis body locations.

Interestingly, when looking at individual AOIs, several lesser-known genes may hold potential for being predictive markers of treatment effect. In a proteomic study, low concentrations of the protein STOM, which is involved in platelet activation, signaling and aggregation, were found to predict the efficacy of methotrexate treatment in psoriasis (28). We observed an upregulation of *STOM* in the epidermis of the LE compared with the scalp suggesting that methotrexate may be more effective in treating psoriasis on the scalp rather than the LE. Furthermore, *B4GALT1* was upregulated in the basal layer of the LE compared to the scalp. *B4GALT1* has been associated with treatment response in ankylosing spondylitis (29). However, its role in psoriasis is unclear. *APOE* and *SOX7* were significantly downregulated in vimentin low areas compared when comparing the scalp with the LE. Although *APOE* polymorphisms may have a pathogenic role in psoriasis, there is insufficient evidence to support its utility as a biomarker during treatment (30). A previous study found the *HOX* family of genes to be related to differences in hyperkeratosis across LS skin from back, abdomen, and extremities, in addition to the location-specific methylation genes *SOX7* in the back, *TNFRSF4* in the extremities and *PDE8A* in the abdomen (17).

Psoriasis is a disease characterized by epidermal hyperplasia and dermal infiltration of immune cells driven by several proinflammatory pathways (31). Interestingly, GSVA suggested a high degree of IL-17 and IL-23 signaling in the epidermis compared with the other AOIs suggesting that more of the inflammatory activity in psoriasis was present in the apical part of the epidermis. We also observed significantly increased IL-23 signaling in LE compared with scalp psoriasis. IL-23 inhibitors have shown high efficacy in treating difficult-to-treat areas, with scalp affection being a marker of rapid effect of IL-23 inhibition (32). Attaining complete skin clearance i.e. absolute PASI 0 during biological treatment is now an attainable goal among patients (33). IL-23 inhibitors, such as guselkumab, can provide benefits for patients with mild psoriasis, and in difficult-to-treat areas. Further research is needed to investigate its effects on patients with shorter disease durations or after extended treatment periods (34). While disease duration may influence treatment response with secukinumab and guselkumab, the efficacy of tildrakizumab, an IL-23 inhibitor, appears unaffected by disease duration (35, 36). Though efforts have been made to identify the correct drug for difficult-to-treat areas such as the scalp, intertriginous areas, and nails, there is a general lack of evidence on the best drug to use for a given area (37). An observational study identified the anterior lower leg, posterior lower leg, elbows, and scalp as recalcitrant treatment sites in patients treated with biologic agents for >6 months (38). The effectiveness of brodalumab, a human monoclonal antibody targeting the IL-17A receptor, has been investigated and was found to be effective in palmoplantar and scalp psoriasis (39).

Interestingly, no association between the Dermatology Life Quality Index and specific locations of recalcitrant psoriasis has been observed, which contrasts with previously established conjecture (14, 38).

By using advanced multiomics techniques like bulk and single-cell RNA-sequencing, researchers may obtain valuable insights into the various gene expression patterns in different skin sites. Techniques such as laser capture microdissection and DSP enable more targeted exploration of disease-affected areas in the skin, by providing gene expression data in a spatial context (40). Though spatial transcriptomics have been utilized in psoriasis (41) this is, to our knowledge, the first study looking at LS skin from different body locations using these emergent technologies.

Disease memory is a challenge in psoriasis and causes relapse in previous areas even after cessation of treatment (8, 11). It has been suggested that this disease memory may be erased by a combination of topical- and systemic treatment, or long-term biological therapy, however this idea warrants further studies (42). TRMs are at heterogenous group of T-cells which may be responsible for this phenomenon as they form a localized long-term memory in epithelial barrier tissues and thus may be the reason behind the observed site-specific treatment resistance in psoriasis (43). However, in the present study, we did not identify a clear association between the differentially expressed genes and established TRM signatures. This suggests that disease persistence may involve a broader cellular or stromal interaction beyond T cells alone. Indeed, recent single-cell and spatial sequencing data have demonstrated that keratinocytes and fibroblasts play a critical role in amplifying inflammatory responses and sustaining the psoriatic microenvironment (44). Future studies using higher-resolution approaches will be essential to determine whether the abundance and functional states of TRMs, or their specific interactions with resident stromal cells as described by Ma et al., differ across anatomical sites to contribute to localized disease persistence.

Regarding histopathological analysis we did not find any significant differences between psoriasis locations. However, it has been reported that the extremities are more prone to hyperkeratosis than the back and abdomen (17).

Limitations of the current study must also be appreciated. First, few patients were included and not all locations could be acquired from all patients. This reduced statistical power—especially for the IHC analyses—and may have contributed to the lack of detectable site-specific differences at the protein level. Further studies should include more patients and more diverse locations. Second, the observed site-specific differences in psoriasis might simply reflect inherent physiological variations across anatomical locations, as previously documented in healthy skin (45). Future studies should include matched location specific non-lesional skin and other skin diseases to better elucidate this. Third, no specific disease severity criteria or plaque-site specific disease severity score were applied. Incorporating standardized, site-resolved severity measures in future work will strengthen clinical–molecular correlations.

In conclusion, we observed transcriptomic differences in different AOIs from different psoriasis locations. Some of the DEGs between different anatomical psoriasis locations here identified may be important for understanding why some treatments are effective in treating psoriasis at specific locations and while others are not.

## Data Availability

The datasets presented in this study can be found in online repositories. The names of the repository/repositories and accession number(s) can be found below: https://www.ncbi.nlm.nih.gov/geo/query/acc.cgi?acc=GSE310145. All other data are available on reasonable request.
