# Supplementary material for: Spatial transcriptomic profiling reveals body site-specific inflammatory differences in psoriasis lesions
Source: Front Immunol. 2026 Mar 12;17:1706701. doi: 10.3389/fimmu.2026.1706701 (PMC13017802; doi:10.3389/fimmu.2026.1706701)
Supplement: Supplementary file 1 [file Supplementaryfile1.docx]

Supplementary Material

# Supplementary Figures and Tables

## Supplementary Figures


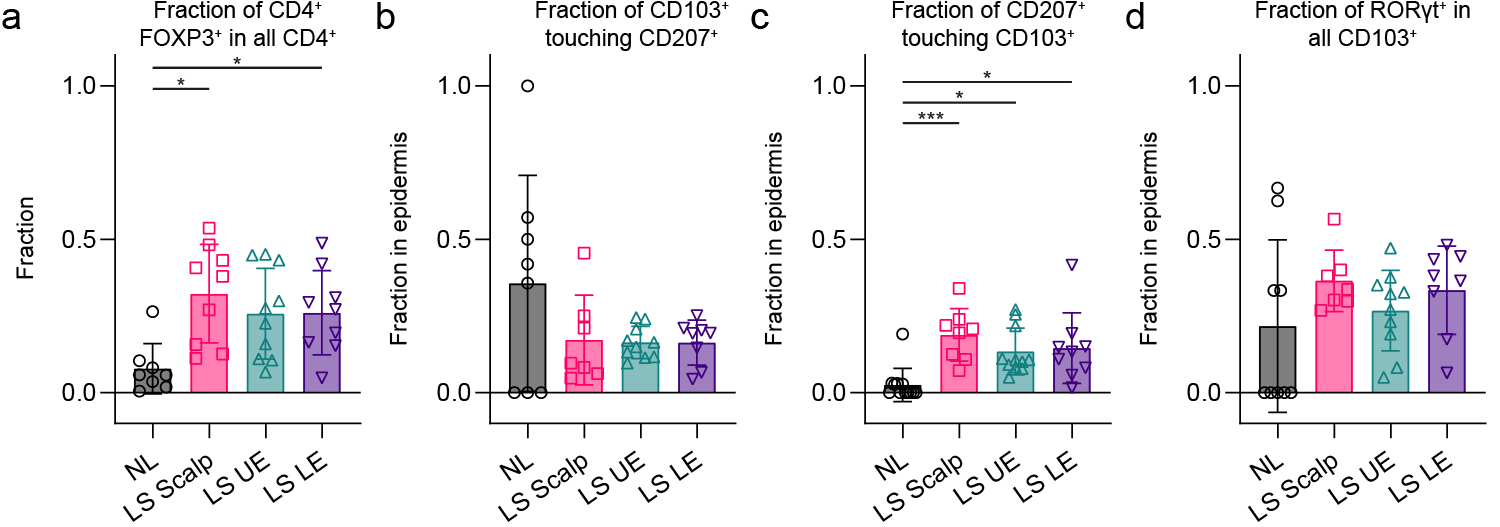


**Supplementary Figure 1.** **Additional immunohistochemical results from different body locations.**

a. Results from quantitative immunohistochemistry of the fraction of CD4^+^ and FOXP3^+^ (double positive cells) in all CD4^+^ cells present in both epidermis and dermis. b. Results from quantitative immunohistochemistry of the fraction of CD103^+^ cells touching a CD207^+^ cell. c. Results from quantitative immunohistochemistry of the fraction of CD207^+^ cells touching a CD103^+^ cell. d. Results from quantitative immunohistochemistry of the fraction of RORγt^+^ in all CD103^+^ cells (double positive cells). Mean ± SD depicted. *P < 0.05, **P < 0.01, ***P < 0.001, ****P < 0.0001.


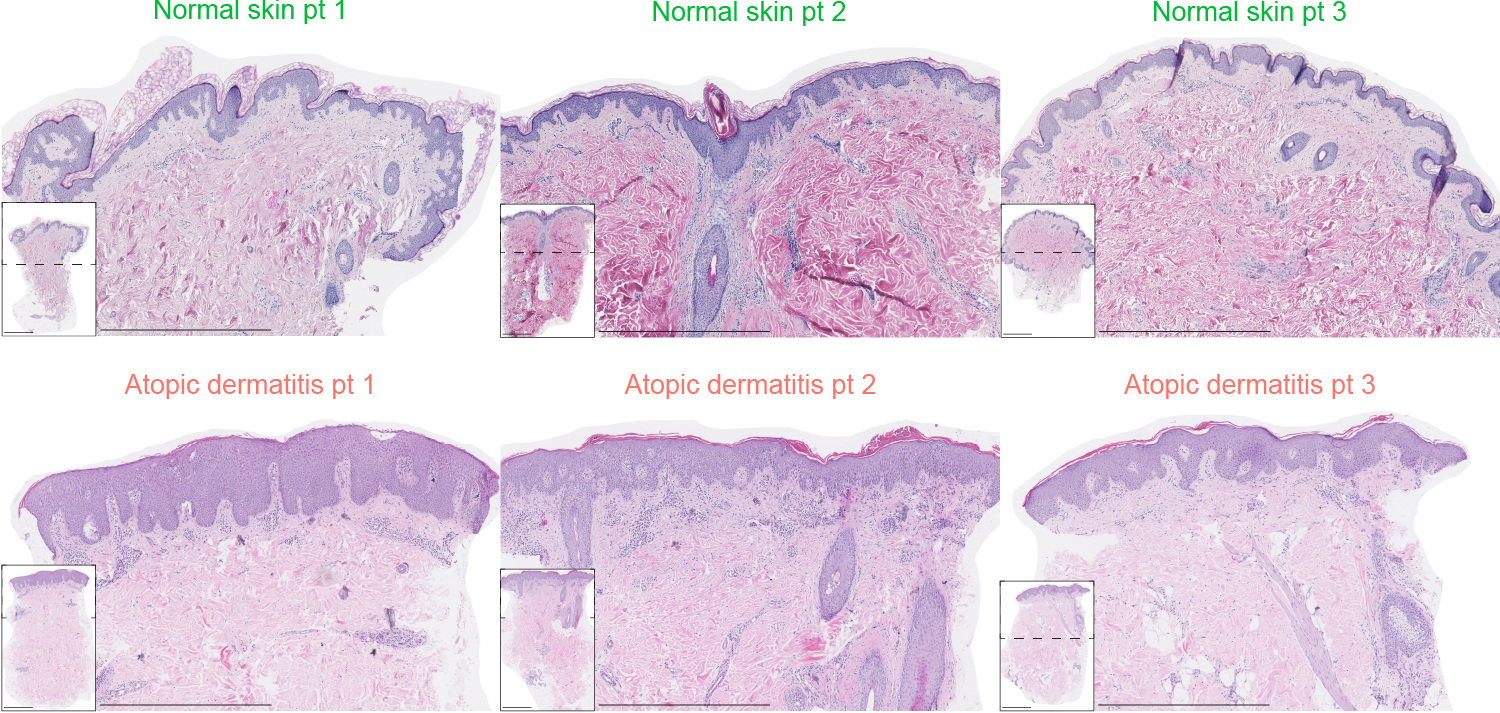


Supplementary Figure 2. Hematoxylin and eosin staining of the three normal skin and atopic dermatitis patients.

Hematoxylin and eosin (HE) staining of the three normal skin and atopic dermatitis samples used for digital spatial profiling analysis. Size bars = 1mm.


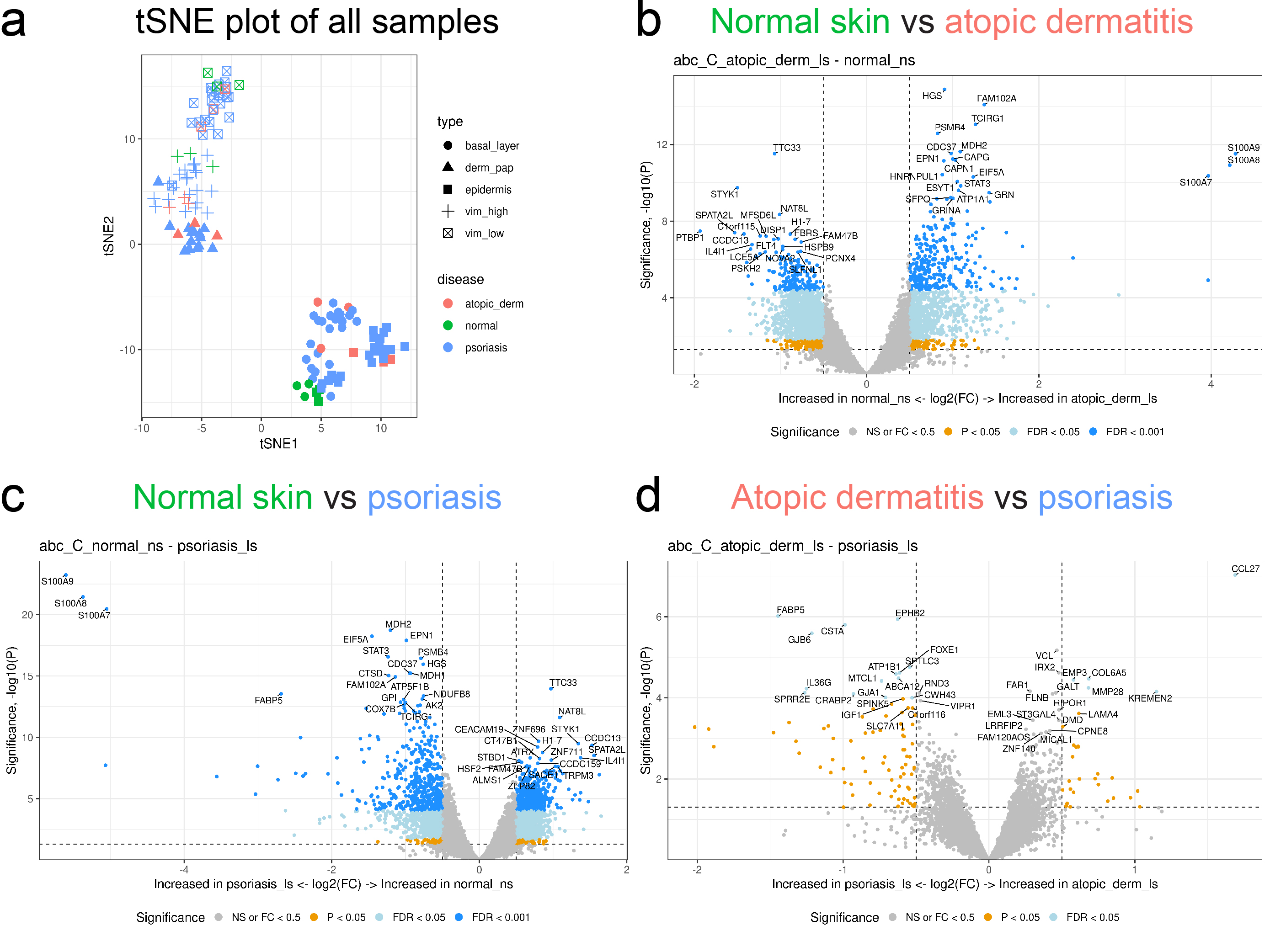


Supplementary Figure 3. Digital spatial profiling of all areas of illumination and skin conditions.

a. tSNE plots of all the samples showing all areas of interest and skin conditions. b. Volcano plot depicting the differentially expressed genes (DEGs) between normal skin and atopic dermatitis of all areas of illumination (AOIs) grouped into one. c. Volcano plot depicting the DEGs between normal skin and psoriasis of all AOIs grouped into one. d. Volcano plot depicting the DEGs between atopic dermatitis and psoriasis grouped into one.


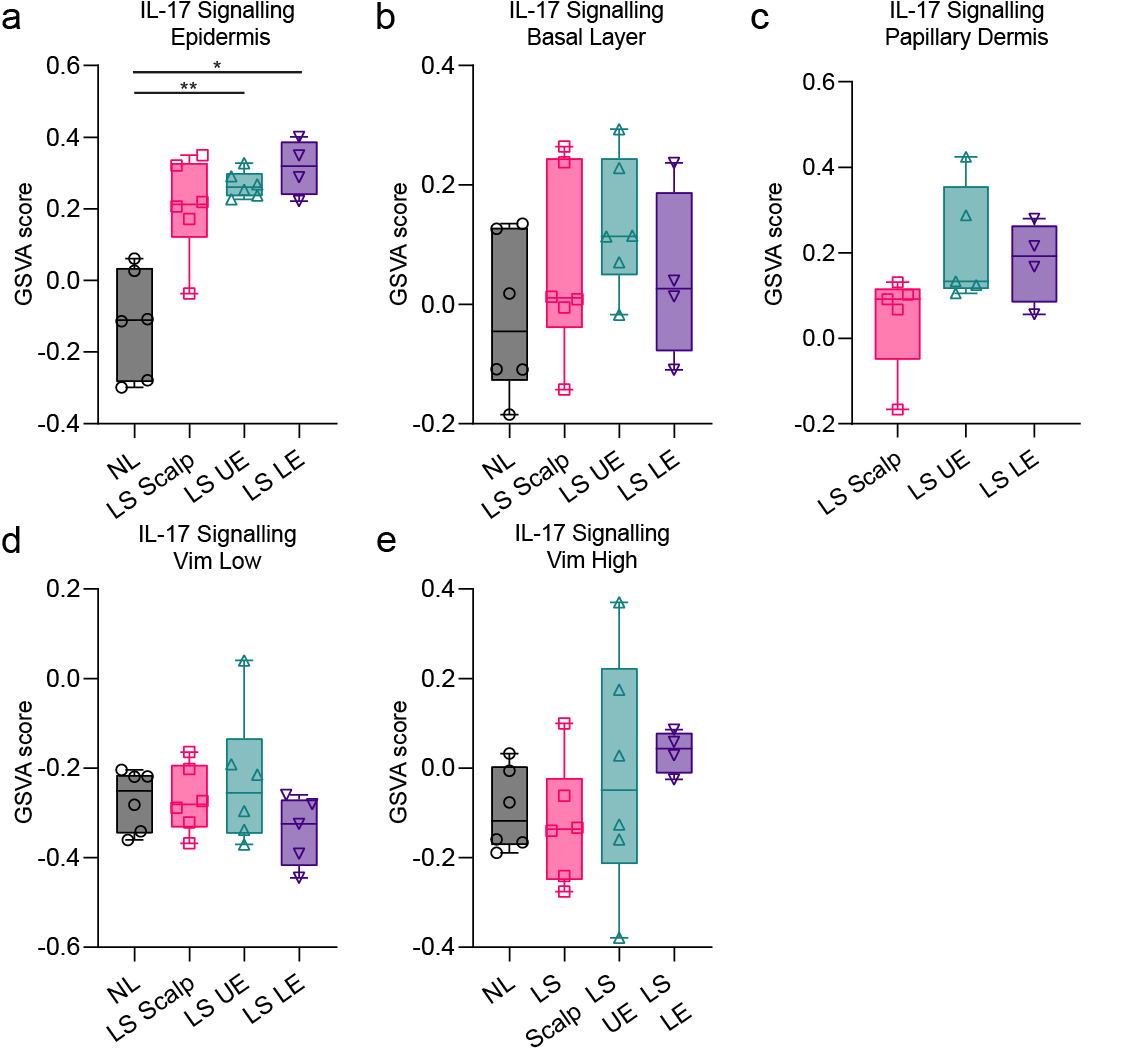


Supplementary Figure 4. Gene set variation analysis of the IL-17 signaling pathway from the different psoriasis body locations.

a. IL-17 signaling in the epidermis. b. IL-17 signaling in the basal layer. c. IL-17 signaling in the papillary dermis. d. IL-17 signaling in the vimentin low (Vim Low) area. e. IL-17 signaling in the vimentin high area (Vim High).


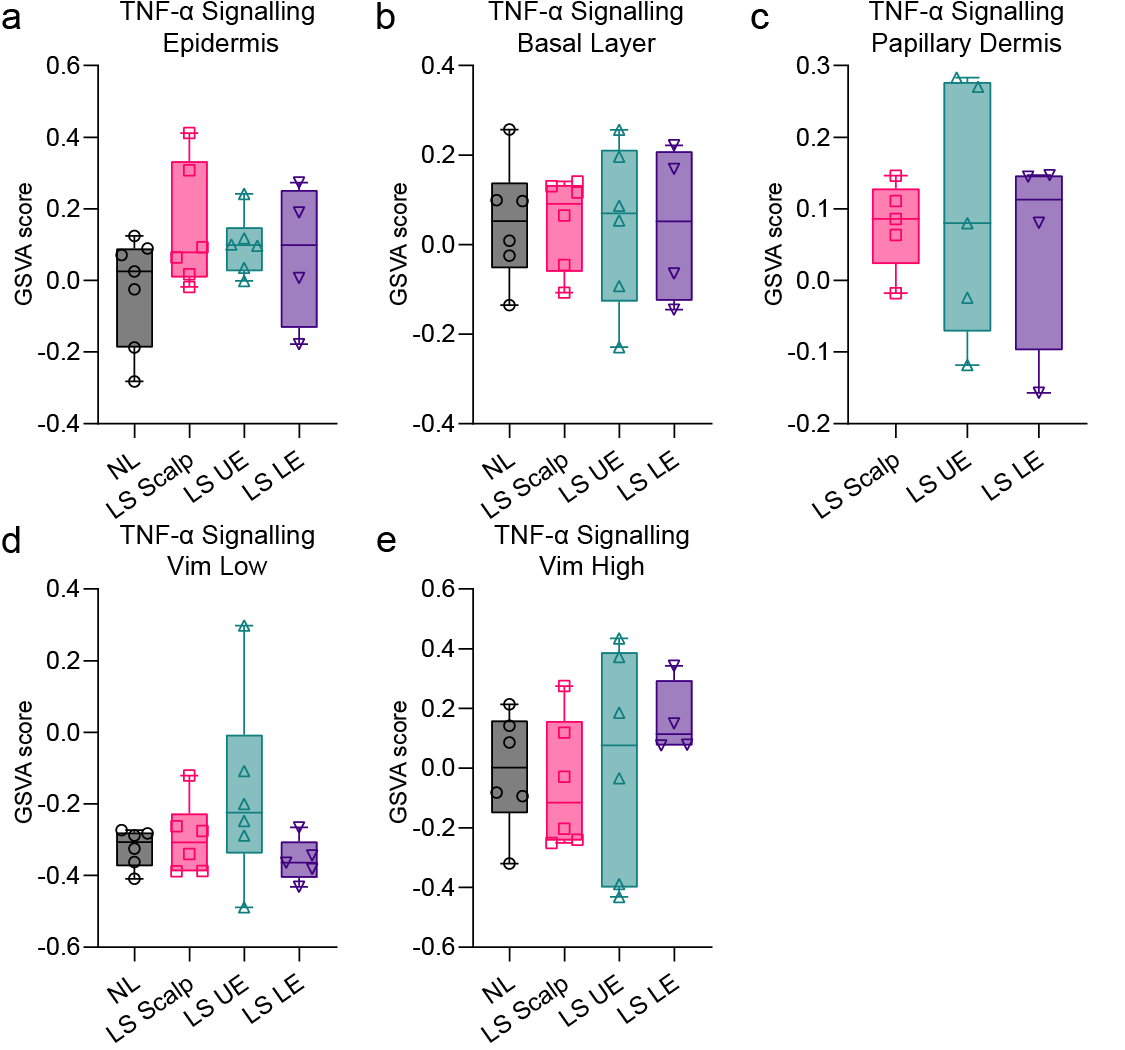


Supplementary Figure 5. Gene set variation analysis of the TNF signaling pathway from the different psoriasis body locations.

a. TNF signaling in the epidermis. b. TNF signaling in the basal layer. c. TNF signaling in the papillary dermis. d. TNF signaling in the vimentin low area (Vim Low). e. TNF signaling in the vimentin high area (Vim High).


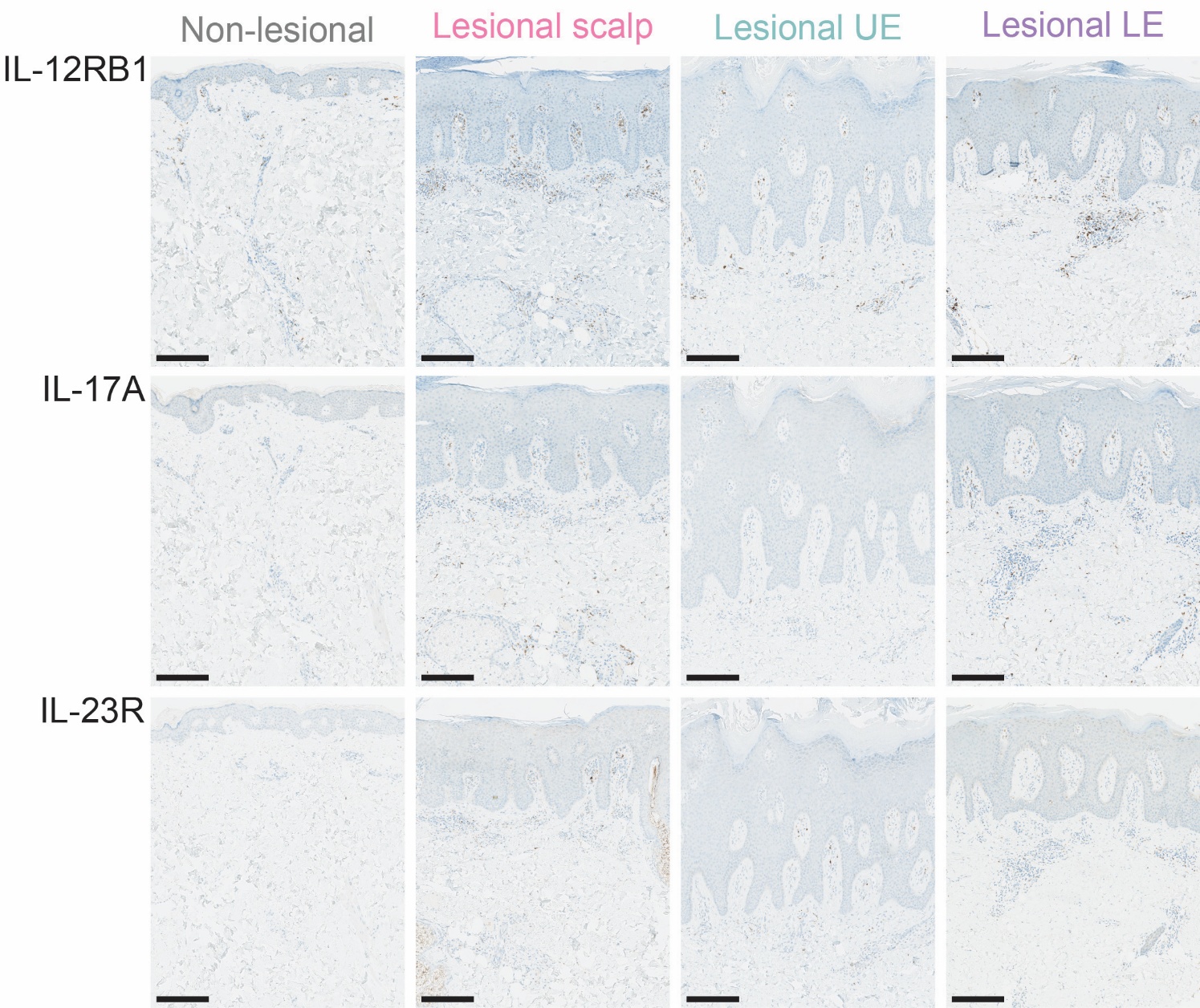


**Supplementary Figure 6. Additional immunohistochemical staining from four body locations.**

Examples of immunohistochemistry staining of IL-12RB1, IL-17A, and IL-23R from non-lesional skin, lesional psoriasis skin from the scalp, lesional skin from the upper extremity (UE), and lesional skin from the lower extremity (LE). Size bars = 250 μm.


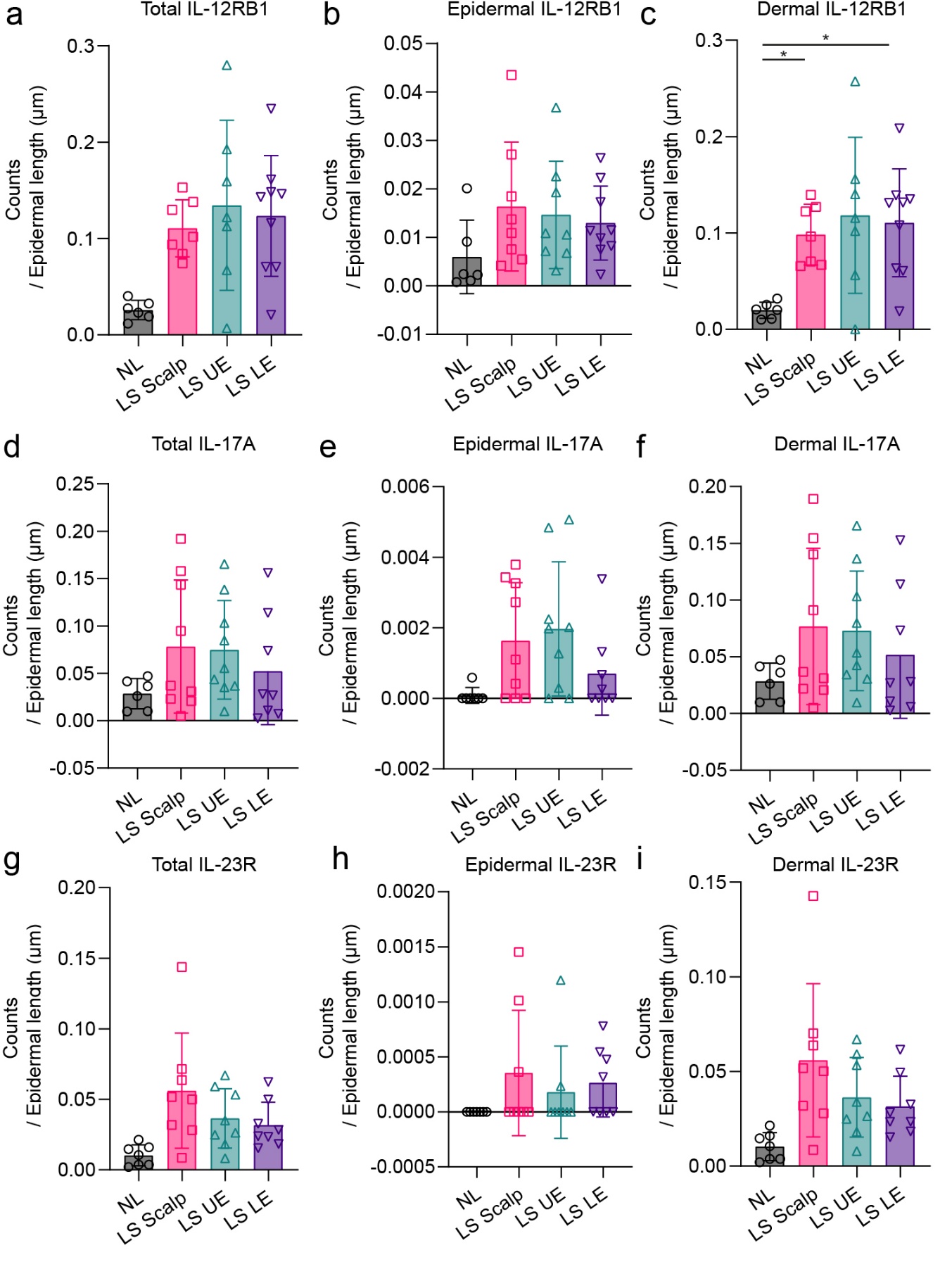


**Supplementary Figure 7. Additional immunohistochemical results from different body locations.**

a-c. Results from quantitative immunohistochemistry of IL-12RB1 as total values and separated into epidermis and dermis. d-f. Results from quantitative immunohistochemistry of IL-17A as total values and separated into epidermis and dermis. g-i. Results from quantitative immunohistochemistry of IL-23R as total values and separated into epidermis and dermis. Mean ± SD depicted. *P < 0.05.

## Supplementary Tables

| **PT#** | **Years** | **Sex** | **Ethnicity** | **Race** | **Duration of Psoriasis (yr)** | **Psoriasis Area and Severity Index** | **Current Treatment (**>**2 weeks prior for topical,** >**4 weeks prior for systemic)** | **Previous treatments for psoriasis** | **Psoriasis Family history** | **Alcohol**  **(**>**10 drinks pr week)** | **Smoking** | **Height (cm)** | **Wight (kg)** | **Body mass index** |
| --- | --- | --- | --- | --- | --- | --- | --- | --- | --- | --- | --- | --- | --- | --- |
| 1 | 61 | M | Danish | White | 1 | NA | None | Dermovate, UVB, Xamiol | None | No | On occasions | NA | NA | NA |
| 2 | 60 | M | Danish | White | NA | NA | NA | NA | NA | NA | NA | NA | NA | NA |
| 3 | 35 | F | Danish | White | NA | 5.1 | None | Methotrexate | None | No | 20 cigarettes daily | NA | NA | NA |
| 4 | 34 | F | Danish | White | 9 | 6 | None | Dermovate, Dead Sea climatotherapy | None | No | None | 165 | 71 | 26.1 |
| 5 | 64 | M | Danish | White | 27 | 3.9 | Dermovate | UVB, Tar, Methotrexate, Neotigason | NA | No | 20 cigarettes daily | 180 | 100 | 30.9 |
| 6 | 30 | M | Danish | White | 10 | 7 | Enstilar, Dermovate | Methotrexate | None | No | Never | 185 | 76 | 22.2 |
| 7 | 31 | M | Danish | White | 23 | 46 | None | Dermovate, Enstilar, UVB, Tildrakizumab | Dad, brother | No | Never | 176 | 76 | 24.5 |
| 8 | 57 | M | Danish | White | 20 | 15 | None | Dermovate, Enstilar, UVB | None | No | Never | 188 | 90 | 25.4 |
| 9 | 41 | F | Danish | White | 20 | 3.2 | Dermovate | Methotrexate, Locoid, Fusidic acid, Betamethasone, Brentacort, Mometasonfuroat | Fathers father, Grandfather, Great grandfather | No | 10 cigarettes daily for 10 years | 176 | 73 | 23.6 |
| 10 | 50 | M | Danish | White | 5 | 12.5 | Enstilar | Dermovate, UVB, Fusicort | Mothers sister, cousin on mothers side | No | 10 cigarettes daily for 40 years | 184 | 82 | 24.2 |
| 11 | 46 | M | Danish | White | 20 | 6 | Locoid | Enstilar, Bucky rays, Methotrexate | Father, daughter | No | Never | 181 | 115 | 35.1 |
| 12 | 37 | M | Danish | White | 25 | 14 | None | Dermovate, Enstilar, UVB, Methotrexate | Sister, mothers father | No | 20 cigarettes daily for 7 years | 167 | 86 | 30.1 |

**Supplementary Table 1. General data of included patients with psoriasis.**

NA = not available, M = male, F = female, UVB = ultraviolet B.

| **PT#** | **Years** | **Sex** | **Ethnicity** | **Race** | **Biopsy location** | **Current Treatment (**>**2 weeks prior for topical,** >**4 weeks prior for systemic)** |
| --- | --- | --- | --- | --- | --- | --- |
| AD01 | 25 | M | Danish | White | NA | None |
| AD02 | 34 | M | Danish | NA | NA | None |
| AD03 | 31 | F | Danish | White | NA | None |
| NS1 | 52 | F | Danish | White | NA | - |
| NS2 | 35 | M | Danish | White | NA | - |
| NS3 | 23 | F | Danish | White | NA | - |

**Supplementary Table 2. General data of included patients with atopic dermatitis and healthy controls.**

AD = atopic dermatitis, NS = normal skin, NA = not available, M = male, F = female.

| **PT#** | **A – Non-lesional skin** | **B – Lesional scalp** | **C – Lesional upper extremity** | **D – Lesional lower extremity** |
| --- | --- | --- | --- | --- |
| PT01 | Abdomen | Scalp | Hand | Ankle |
| PT02 | Shoulder | **No sample** | Finger | **No sample** |
| PT03 | Abdomen | **No sample** | Arm | Lower leg |
| PT04 | Abdomen | Scalp | **No sample** | Ankle |
| PT05 | Abdomen (WTA) | Scalp (WTA) | Elbow (WTA) | **No sample** |
| PT06 | Shoulder (WTA) | Scalp (WTA) | Elbow (WTA) | Ankle (WTA) |
| PT07 | Shoulder (WTA) | Scalp (WTA) | Hand (WTA) | Ankle (WTA) |
| PT08 | Abdomen (WTA) | Scalp (WTA) | Hand (WTA) | Ankle (WTA) |
| PT09 | Abdomen | **No sample** | Hand | Ankle |
| PT10 | Abdomen (WTA) | Scalp (WTA) | Hand (WTA) | Ankle (WTA) |
| PT11 | Abdomen | **No sample** | Elbow | **No sample** |
| PT12 | Abdomen (WTA) | Scalp (WTA) | Arm (WTA) | Ankle (WTA) |

**Supplementary Table 3. Locations of skin biopsies taken from each patient with psoriasis.**

The table displays the sites of skin biopsies taken from each patient, as well as those utilized for whole transcriptome analysis (WTA) using digital spatial profiling.

| Marker | Cell/Target | Clone | Lot | Dilution | Incubation length | Vendor | Isotype | Cat Number | RRID | Flourophore |
| --- | --- | --- | --- | --- | --- | --- | --- | --- | --- | --- |
| CD3 | T-cell | MRQ-39 | 44612 | 1:200 | 30 min | Cell Marque | Rabbit Monoclonal, IgG_1_ | 103R-96 | Nolan lab – Stanford Cat# 103R, RRID:AB_2864399 | - |
| CD4 | Helper T-cell | ZM180 | TP28554F-A | 1:25000 | Overnight | Zeta Corporation | Mouse Monoclonal, IgG_2a/κ_ | Z2482MT | Zeta Corporation Cat# Z2482M, RRID:AB_2892657 | - |
| CD8 | Cytotoxic T-cell | SP16 | 51708 | 1:500 | 30 min | Cell Marque | Rabbit Monoclonal, IgG_1_ | 108R-16 | Cell Marque Cat# 108R, RRID:AB_2892088 | - |
| CD103 | Skin resident cell | EP206 | 105295 | 1:60 | 30 min | Cell Marque | Rabbit Monoclonal, IgG | 437R-15 | Cell Marque Cat# 437R, RRID:AB_2884943 | - |
| CD207 | Langerhans cell | 12D6 | 70437 | 1:100 | 30 min | Cell Marque | Mouse Monoclonal, IgG_2b/κ_ | 392M-15 | Millipore Cat# 392M-1, RRID:AB_2889342 | - |
| FOXP3 | Regulatory cell | EP340 | 20082419 | 1:90 | 30 min | Cell Marque | Rabbit Monoclonal, IgG | AC-0304RUOC | Cell Marque Cat# AC-0304RUO, RRID:AB_2892654 | - |
| IL-12RB1 | Cytokine signaling | EPR23693-37 | - | 1:100 | 30 min | Abcam | Rabbit Monoclonal, IgG | ab256805 | Abcam Cat# ab256805, RRID:AB_3697686 | - |
| IL-17A | Proinflammatory cytokine | - | - | - | 30 min | R&D Systems | Goat Polyclonal, IgG | AF-317-NA | R and D Systems Cat# AF-317-NA, RRID:AB_354463 | - |
| IL-23R | Immune response cytokine | EPR22838-4 | - | - | 30 min | Abcam | Rabbit Monoclonal, IgG | ab222104 | RRID: AB_3697685 | - |
| Ki67 | Proliferation | SP6 | 62103 | 1:200 | 30 min | Cell Marque | Rabbit Monoclonal, IgG | 275R-16 | Cell Marque Cat# 275R-16, RRID:AB_1158037 | - |
| MPO | Neutrophil | SP72 | 46344 | 1:225 | Overnight | Cell Marque | Rabbit Monoclonal, IgG_1_ | 289R-14 | Cell Marque Cat# 289R, RRID:AB_2892655 | - |
| RORγt | Th17 cell | 6F3.1 | 3728331 | 1:3300 | 30 min | Sigma-Aldrich | Mouse Monoclonal, IgG_2a/κ_ | MABF81 | Millipore Cat# MABF81, RRID:AB_11205416 | - |
| PanCK | Epithelial | AE1+AE3 | 211022-01 | As provid-ed by NanoS-tring | 60 min | Novus | Mouse Monoclo-nal, IgG_1/κ_ /IgG1 Kappa | NBP2-33200 | Novus Cat# NBP2-33200AF532, RRID:AB_2924722 | Alexa Fluor® 532 |
| DNA | Nuclei | Syto13 | - | As provided by NanoString | 60 min | NanoString | - | As provided by NanoString | - | Alexa Flour® 525 |
| Vimentin | Filament protein and mesenchymal cells | - | - | As provided by NanoString | 60 min | NanoString | - | As provided by NanoString | - | Cy5 |

**Supplementary Table 4. List of antibodies used for immunohistochemistry and digital spatial profiling.**

†, CD, cluster of differentiation; FOXP3, forkhead box P3; IL, interleukin; MPO, myeloperoxidase; RORγt, RAR-relateret orphan receptor gamma; RRID, research resource identifier.

**Supplementary Table 5. List of reactome genesets used for the different pathways.**
